# Expressed Repeat Elements Improve RT-qPCR Normalization across a Wide Range of Zebrafish Gene Expression Studies

**DOI:** 10.1371/journal.pone.0109091

**Published:** 2014-10-13

**Authors:** Suzanne Vanhauwaert, Gert Van Peer, Ali Rihani, Els Janssens, Pieter Rondou, Steve Lefever, Anne De Paepe, Paul J. Coucke, Frank Speleman, Jo Vandesompele, Andy Willaert

**Affiliations:** Center of Medical Genetics, Ghent University, Ghent, Belgium; Karlsruhe Institute of Technology, Germany

## Abstract

The selection and validation of stably expressed reference genes is a critical issue for proper RT-qPCR data normalization. In zebrafish expression studies, many commonly used reference genes are not generally applicable given their variability in expression levels under a variety of experimental conditions. Inappropriate use of these reference genes may lead to false interpretation of expression data and unreliable conclusions. In this study, we evaluated a novel normalization method in zebrafish using expressed repetitive elements (ERE) as reference targets, instead of specific protein coding mRNA targets. We assessed and compared the expression stability of a number of EREs to that of commonly used zebrafish reference genes in a diverse set of experimental conditions including a developmental time series, a set of different organs from adult fish and different treatments of zebrafish embryos including morpholino injections and administration of chemicals. Using geNorm and rank aggregation analysis we demonstrated that EREs have a higher overall expression stability compared to the commonly used reference genes. Moreover, we propose a limited set of ERE reference targets (*hatn10*, *dna15ta1* and *loopern4*), that show stable expression throughout the wide range of experiments in this study, as strong candidates for inclusion as reference targets for qPCR normalization in future zebrafish expression studies. Our applied strategy to find and evaluate candidate expressed repeat elements for RT-qPCR data normalization has high potential to be used also for other species.

## Introduction

Reverse transcription quantitative PCR (RT-qPCR) is currently regarded as the gold standard for efficient measurement of mRNA gene expression, especially because of its high sensitivity, specificity, accuracy and precision, but also because of its practical simplicity and processing speed. However, variable yields of RNA extraction and reverse transcription and also variable amplification efficiencies can affect RT-qPCR results [1], [2]. To correct for technically induced variation and thus measure true biological variation in samples, it is important to apply a good normalization strategy. The use of multiple reference genes as internal controls is the most frequently applied and recommended procedure for normalizing RT-qPCR data [3]–[7]. In this respect, specific attention should be given to the correct selection and validation of reference genes for normalization, as stated in the MIQE (Minimum Information for Publication of Quantitative Real-Time PCR Experiments) guidelines [1]. The selected reference genes should be stably expressed in the studied samples and should thus show a strong correlation with the total amount of mRNA present in the samples. Importantly, many commonly used reference genes are not generally applicable as their expression stability greatly varies under different experimental conditions [8]–[11]. Therefore, it is essential to determine the optimal number and choice of reference genes for the specific experimental conditions in every study. A number of studies have measured and compared the expression stability of a set of commonly used reference genes in samples derived from different species, organs, cells, developmental stages, and treatments, using one of the available tools that automatically calculate expression stability values (geNorm, BestKeeper, Normfinder) [8]–[11]. These studies propose the set of most stably scored reference genes as being the most suitable for normalizing gene expression data. However, the determination of stable reference genes only occurs in a comparative fashion and the detection of the ‘most stably’ expressed genes does not necessarily mean they are stably expressed in other conditions. Especially developmental time series and the comparison of different tissues are challenging experimental conditions to normalize [8], [11], [12]. Therefore, the ideal situation of using only one set of reference genes to cover all experimental conditions in a specific species has not been feasible up to now.

To tackle the aforementioned issues, we build upon a new concept, first proposed for human samples [13]–[15]. This novel normalization method uses expressed repetitive elements (ERE) as reference targets, instead of protein coding mRNAs. Here, we illustrate the usefulness of this approach for zebrafish expression data. The zebrafish (*Danio rerio*), a small teleost fish, is a popular vertebrate model organism for a number of reasons, including the low maintenance cost, short reproductive cycle, external fertilization and development, production of large numbers of synchronous and rapidly developing embryos per mating and the optical transparency of zebrafish embryos. Moreover, the availability of a wide range of molecular techniques, such as overexpression/knockdown approaches, transgenesis, large-scale genome mutagenesis and lately also highly efficient targeted mutagenesis (using ZFN, TALEN and CRISPR-Cas technology) make zebrafish an excellent tool for high-throughput disease modeling. Finally, molecular genetic mechanisms and cellular physiology are highly similar between zebrafish and other vertebrates, underscoring the relevance of zebrafish for the modeling of human diseases.

We assessed and compared the expression stability of a number of EREs in the zebrafish transcriptome to a set of commonly used zebrafish reference genes in a developmental time series, in different organs from adult fish and under different treatments of zebrafish embryos including morpholino injections and administration of chemicals. Here we demonstrate that EREs outperform classically used reference genes and put forward a selection of EREs as strong candidates for inclusion as reference targets for qPCR normalization in a diverse set of zebrafish experiments. The procedure followed here for identification of zebrafish reference EREs can also easily be applied for other species.

## Materials and Methods

### Zebrafish maintenance and imaging

Wild-type AB zebrafish, obtained from the zebrafish international resource center (ZIRC) were maintained in 3.5 liter tanks in Zebtec semi-closed recirculation housing systems (Tecniplast, Italy) at a constant temperature of 28°C and a 14 h light 10 h dark photoperiod. Fish were fed 4 times a day with both dry feed (SDS, UK) and brine shrimps (Ocean Nutrition, Belgium). After *in vitro* fertilization, dead embryos were removed at 8 hpf (hours post fertilization) and at 24 hpf surviving embryos were dechorionated with pronase (Sigma, St. Louis, MO, USA). At 48 hpf or 72 hpf, embryos were anesthetized with 0.016% tricaine methanesulfonate (tricaine) and mounted in 2% methylcellulose and imaged using a Leica M165FC stereomicroscope. Approval for this study was provided by the local committee on the Ethics of Animal Experiments (Ghent University Hospital, Ghent, Belgium; Permit Number: ECD 11/37). All efforts were made to minimize pain and discomfort.

### Morpholino injections

Morpholinos (MOs) are small antisense oligonucleotides that bind the mRNA of interest, resulting in a down regulation of the gene expression. In this screen, MOs targeting *chordin and slc2a10* were injected. A scrambled MO was also included as a negative control. *Chordin* encodes for a secreted protein that dorsalizes early vertebrate embryonic tissues and is often used as a positive control in MO experiments [16]. Chordin-MO injected embryos display abnormal u-shaped somites, an expanded blood island and an abnormal tail fin with multiple folds. *Slc2a10* encodes for GLUT10, a member of the glucose transporter family. Recessive mutations in this gene are causing the arterial tortuosity syndrome (ATS) [17]. In zebrafish embryos, knockdown of *slc2a10* using MO injection causes a wavy notochord and cardiovascular abnormalities with a reduced heart rate and blood flow, which was coupled with an incomplete and irregular vascular patterning [18]. Morpholino oligonucleotides were obtained from Gene Tools, LLC (Philomath, OR, USA). The MO against *slc2a10* (5′-CAAATAAAGTCCACTTACTTGGTCC-3′) is directed against the exon 2–intron 2 donor splice site of the *slc2a10* pre-mRNA [18]. For *chordin,* the MO is directed against the start codon (5′-ATCCACAGCAGCCCCTCCATCATCC–3′) [16]. A control MO (5′-CCTCTTACCTCAGTTACAATTTATA-3′) was used as a negative control in each experiment. MOs were microinjected in 1.5 nl volume into 1- to 2-cell stage embryos at 7.5 ng for *slc2a10*, 2 ng for *chordin*, and 5 ng for the control MO. All MOs were dissolved in 0.1% phenol red and 1× Danieu’s buffer [58 mM NaCl, 0.7 mM KCl, 0.4 mM MgSO4, 0.6 mM Ca(NO3)2, 5.0 mM HEPES (pH 7.6)]. Microinjection procedures were performed using a Leica M80 stereomicroscope. At 48 hpf, embryos were dechorionated, euthanized with 0.4% tricaine, and triplicate pools of 20 embryos were collected in RNAlater (Sigma-Aldrich, St. louis, USA).

### Compound treatments

Two different chemical treatments were performed: embryos were treated with 40 µM of TGFβ type 1 receptor kinase inhibitor (TGFBRI, LY-364947, #L6293, Sigma, St. Louis, USA), or 194 µM of warfarin (Coumadin, #45706 Sigma, St. Louis, USA). TGFBRI specifically targets the TGFBR1 kinase function resulting in the inhibition of phosphorylation of SMAD2 and SMAD3 and down regulation of TGFβ signaling. Treatment of early embryos with this inhibitor results in cardiovascular abnormalities including condensation of the caudal vein plexus, low heart rate and reduced blood flow [18]. Warfarin, is an oral anticoagulant drug used in treatment of thromboembolic diseases [19]. Warfarin acts as a vitamin K antagonist, and vitamin K is needed as a cofactor for the carboxylation of glutamate residues of several clotting factors. Administration of warfarin to early embryos produces teratogenic effects including developmental delay, growth retardation, eye defects, scoliosis and ear defects [20].

TGFBRI and warfarin were prepared as a 20 mM and 80 mM stock solution respectively in DMSO. Working solutions, 0 and 40 µM for TGFBRI and 0 and 194 µM for warfarin, were made in E3 chemical screening medium [21] and as previously described [18], [20], embryos were incubated in the compounds starting at 8 hpf (TGFBRI) and 2.5 hpf (warfarin), dechorionated at 24 hpf, euthanized with 0.4% tricaine, and collected in triplicate pools of 20 embryos in RNAlater at 48 hpf (TGFBRI) or 72 hpf (warfarin).

### Developmental time series embryos/larvae and dissection of organs from adult zebrafish

At several time points (0 hpf, 8 hpf, 24 hpf, 48 hpf, 72 hpf, 96 hpf, 6 dpf, 8 dpf, 10 dpf and 12 dpf), triplicate pools of 20 embryos/larvae were collected, euthanized with 0.4% tricaine, and stored in RNAlater. Dissection of the eye, brain, skin, testis, liver, intestines and ovaria from two adult fish was performed as previously described [22]. After dissection, the organs were immediately snap frozen using liquid nitrogen. Subsequently they were sectioned (50 µm) using a Leica CM1900 cryotome and lysed in 700 µl of Qiazol (Qiagen, Germantown, USA).

### RT-qPCR

RT-qPCR reactions were performed and reported according to MIQE guidelines [1]. If needed, RNAlater was first removed from samples with a glass Pasteur pipette and RNA isolation was performed using the miRNeasy mini kit (Qiagen) in combination with on-column DNase I treatment using the RNase-Free DNase set (Qiagen) according to the manufacturer’s guidelines. RNA quality index (all RQI>8) was measured for all the samples using an Experion automated electrophoresis system (software version 3.2, Bio-Rad). As the RNA concentration of the adult tissue samples was low, whole transcriptome amplification for these samples was executed as previously described (NuGEN) [23]. cDNA was synthesized from 1 µg RNA in a 20 µl reaction with the iScript kit (Bio-Rad) using a blend of oligodT and random hexamer primers. qPCR reactions were performed in a total volume of 5 µl, comprising 2.5 µl SsoAdvanced SYBR Green Supermix (Bio-Rad), 5 ng (total RNA equivalents) cDNA and 250 nM (final concentration) of each primer on a LightCycler 480 qPCR instrument (Roche) in 384-well white plates (Bio-Rad). Thermocycling conditions were as follows: 95°C for 2 min, followed by 44 cycles of 95°C for 5 s, 60°C for 30 s, 72°C for 1 s and finally a melting curve analysis was performed at 95°C for 5 s followed by 60°C for 1 min, gradual heating to 95°C at a ramp-rate of 0.11°C/s followed by cooling to 37°C for 3 min. Primers for *bactin2*, *elfa*, *cyp19a1b*, *hprt1*, *rps18*, *tbp*, *rpl13a*, *tuba1* and *b2m* were designed using primerXL software (http://primerxl.org/). Primer sequences for *gapdh* were taken from literature [11]. Primers for the newly identified expressed repeats were designed with primer3 software (http://primer3.ut.ee/) using default settings [24]. Primer efficiencies were tested using a standard dilution series: RNA extracted from different developmental stages of zebrafish embryos (8, 24, 30, 48, 72, 96 hpf) was pooled and converted to cDNA to make a standard dilution series ranging from 16 ng to 0.0625 ng (Figure S1A). Primer specificity was evaluated using melt-curve analysis (Figure S1B). Primer efficiencies were also determined using LinRegPCR software [25]. For this, the raw, non-baseline-corrected qPCR data were exported from the LightCycler 480 software and imported into the LinRegPCR software. A complete overview of all primer sequences and concomitant PCR efficiencies used in this study can be found in Table 1 and S1.

**Table 1:** Reference target primer design and calculation of amplification efficiencies.

Reference target (*)	Forward primer	Reverse primer	Amplification efficiency (%)	Primer design
*tc1n1*	TGTCTGGGTTGGTGTTGTAT	GCTCTGTCGACTTTTGATGT	103.5	Primer3 [22]
*dna11ta1*	GGGACAACATGAAGGAATTGT	AAAAATGCAGGGTTCCACACA	107.6	Primer3 [22]
*tdr7*	GCAGCATAATTGAGTACACCC	TTGCCTATATTCACTGAGAAATGGA	102.2	Primer3 [22]
*dna15ta1*	TACTGTGCTCAAATTGCTTCA	AATGAGTACTGTGAACTTAATCCAT	101.1	Primer3 [22]
*cr1-1*	GCTCTTCAGTGTTTGAACTCTCAGT	CAATGTAGATTGTGTCAAAGCAG	101.2	Primer3 [22]
*hatn8*	CAATGACGGTTGGGGTTAGG	TTTAAAAAGGAGGCGTGGCA	102.4	Primer3 [22]
*hatn10*	TGAAGACAGCAGAAGTCAATG	CAGTAAACATGTCAGGCTAAATAA	104.3	Primer3 [22]
*hatn4*	ACCCTGATCAAACACACCTG	TCAAGTGCTGTTCAGGTCCTA	105.0	Primer3 [22]
*loopern4*	TGAGCTGAAACTTTACAGACACAT	AGACTTTGGTGTCTCCAGAATG	109.5	Primer3 [22]
*sine3*	GGAGACCACATGGGAAAACT	AGAGTCAGGACCTCGGTTTA	101.4	Primer3 [22]
*tuba1*	TCATCTTCTCCTTCCACACT	GTACGTGGGTGAGGGTAT	107.7	PrimerXL
*tbp*	AAGTTTACGGTGGACACAAT	CAGGCAACACACCACTTTAT	95.4	PrimerXL
*b2m*	ACGCTGCAGGTATATTCATC	TCTCCATTGAACTGCTGAAG	94.6	PrimerXL
*elfa*	GGAGACTGGTGTCCTCAA	GGTGCATCTCAACAGACTT	106.0	PrimerXL
*cyp19a1b*	AAGGCCATCCTAGTAACCAT	GGTTGTTGGTCTGTCTGATG	101.5	PrimerXL
*bactin2*	ACGATGGATGGGAAGACA	AAATTGCCGCACTGGTT	99.3	PrimerXL
*rpl13α*	AGGCTGAAGGTGTTTGATG	TTTCAGACGCACAATCTTGA	91.2	PrimerXL
*hprt1*	GAGGAGCGTTGGATACAGA	CTCGTTGTAGTCAAGTGCAT	95.7	PrimerXL
*rps18*	AGTTCTCCAGCCCTCTTATT	TCAACACGAACATTGATGGA	98.7	PrimerXL
*gapdh*	GTGGAGTCTACTGGTGTCTTC	GTGCAGGAGGCATTGCTTACA	102.5	McCurley [11]

(*)HUGO or repbase identifier.

### Statistics and data analysis

The geNorm module in qbase^+^ version 2.5 (Biogazelle, http://www.qbaseplus.com) was used to compute expression stability values for all reference targets. As input for geNorm analysis, either Cq values exported directly from the LightCycler 480 software or efficiency-corrected Cq values from LinRegPCR that were calculated based on the raw, non-baseline-corrected LightCycler 480 qPCR data, were used. GeNorm calculates the gene expression stability measure M (M-value) for a reference gene as the average pairwise variation V for that gene with all other tested reference genes. Stepwise exclusion of the gene with the highest M value allows ranking of the tested genes according to their expression stability. GeNorm was also used to dertermine the optimal number of reference targets for every experiment. The geNorm algorithm determines the pairwise variation Vn/n+1, between two sequential normalization factors containing an increasing number of genes. A large variation means that the added gene has a significant effect and should preferably be included for calculation of a reliable normalization factor. Vandesompele *et al.* (2002) [7] used 0.15 as a cut-off value, below which the inclusion of an additional reference gene is not required.

Rank aggregation analysis was performed in the R statistical programming environment (version 3.0.2) using the Rankaggreg package (version 0.4–3) [26] to determine the best ranked reference genes across all experiments.

## Results

### Identification of candidate expressed repeat element (ERE) reference targets in the zebrafish genome

**Figure 1:**
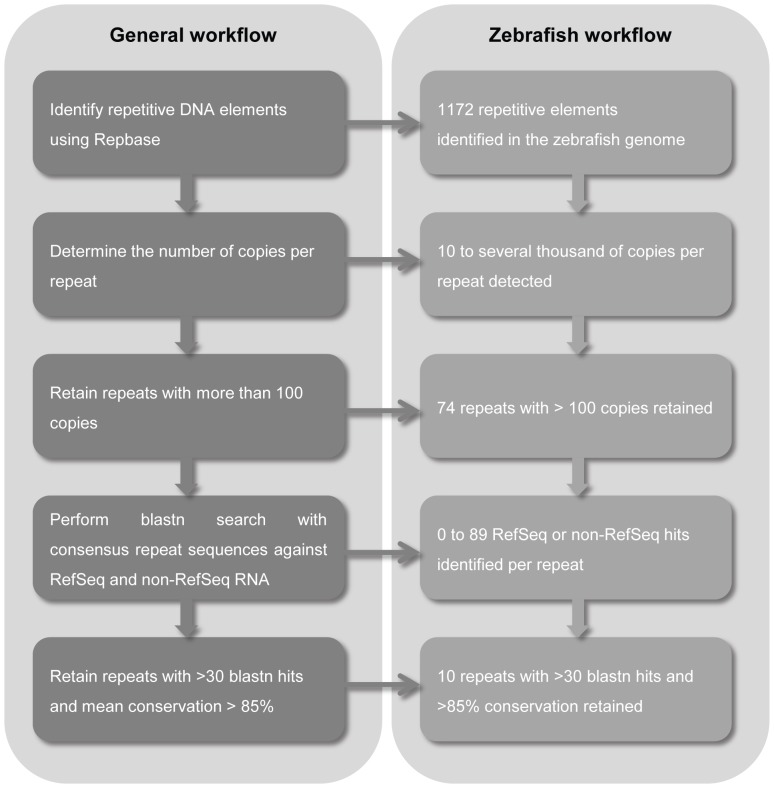
Workflow to identify candidate expressed repeat elements.

Candidate ERE reference targets in the zebrafish genome were extracted from Repbase (http://www.girinst.org/repbase), a database of repetitive DNA elements from different organisms [27] (Figure 1). From an initial set of 1172 repetitive elements present in the zebrafish genome, only those having more than 100 copies in the genome were retained, leaving us with 74. To identify the number of expressed loci per repetitive element, a blastn search against all RefSeq and non-RefSeq annotated transcripts known for zebrafish was carried out using the consensus repeat sequence listed in Repbase. Only repeats with a total number of combined RefSeq and non-RefSeq blast hits above 30 and with a mean conservation rate higher than 85% (indicated by Repbase) were retained, resulting in 10 candidate EREs for further analysis (*tc1n1, dna11ta1, tdr7, dna15ta1, cr1-1, hatn8, hatn10, hatn4, loopern4, sine3*). The thresholds of 30 and 85% were empirically determined in order to have a top-ranked list containing a manageable number of candidate expressed repeat elements. Next, qPCR assays were designed to target the most conserved region of the selected EREs (Table 1 and Figure S2). Blasting of the primer sequences against the zebrafish RefSeq RNA database using primer-BLAST (http://www.ncbi.nlm.nih.gov/tools/primer-blast/) revealed that the amplified ERE fragments are exclusively located in untranslated gene regions, predominantly 3′UTR.

To investigate the potential of EREs for qPCR normalization, we aimed to compare the expression stability of the 10 candidate EREs with that of 10 commonly used reference genes in zebrafish studies. The reference genes *bactin2*, *elfa*, *cyp19a1b*, *hprt1*, *rps18*, *tbp*, *rpl13a*, *tuba1*, *b2m* and *gapdh* were selected because of their frequent use in zebrafish expression studies. The amplification efficiency of all primer pairs was assessed using a zebrafish cDNA dilution series as a template, wherein efficiencies between 90 and 110% were attained indicating sufficient reaction efficiencies (Table 1 and S1).

### Determination of reference target expression stabilities under a wide range of conditions

**Figure 2:**
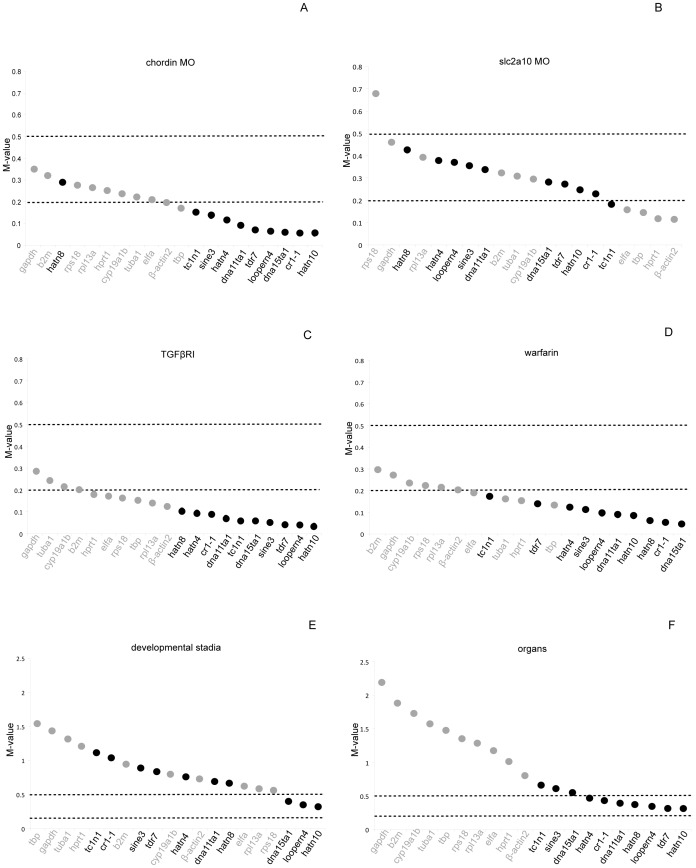
Average expression stability of common reference genes and expressed repeat elements. Ranking of reference targets depending on their M-values calculated by geNorm. Reference targets with M-values below 0.5 and 0.2 are considered having a ‘high’ and ‘very high’ expression stability, respectively. EREs are indicated in black, commonly used reference mRNAs in grey.

For the 20 candidate reference targets (10 EREs and 10 commonly used reference genes) mRNA expression levels were measured in a wide range of experimental settings including a zebrafish developmental time series (0 hpf up to 12 dpf), a set of different organs dissected from adult fish and a set of different treatments of zebrafish embryos including the administration of chemicals and injection of morpholinos (MO) (see Methods). The average expression stability for each of the reference targets in the 4 different types of experiments was calculated using the geNorm algorithm. Reference genes are ranked according to their expression stability value (referred to as the M-value) [7]; in addition, the optimal number of genes for normalization is determined for each experiment. Reference targets with M-values below 0.5 and 0.2 are considered having a ‘high’ and ‘very high’ expression stability, respectively [12]. In the experiments where embryos were treated with compounds or injected with MOs almost all reference targets had a ‘high’ expression stability and a considerable number of reference targets showed a ‘very high’ expression stability (Figure 2A–D). In general, the EREs showed higher expression stabilities (lower M-values) compared to the reference genes, although differences in M-values are small. In the developmental time series and the comparison of the different zebrafish organs, the M-value distribution was more dispersed with relatively low expression stability (M>0.5) for the reference genes and ‘high’ to ‘very high’ expression stability for a considerable number of EREs (Figure 2E,F). In the time series, the ERE *hatn10,* was identified as the best reference target, with an M-value around 0.3, while the best performing mRNA reference gene was *rps18* with an M-value around 0.6 (Figure 2E). Of note, *gapdh*, a frequently used reference target in zebrafish, had an M-value of 1.5, which is considered as highly unstable. In the different zebrafish organs (Figure 2F) the best reference target is the ERE *hatn10*, with an M-value of 0.3, while the best classically used reference gene, *bactin2*, had an M-value of only 0.8. Similar results were obtained by performing a geNorm analysis for the 6 different experiments, using efficiency-corrected Cq values that were determined by linear regression analysis of qPCR fluorescence data using LinRegPCR software (Figure S3) [25]. To determine the optimal number of reference targets to be used in the different experiments, the V_n/n+1_ value was calculated using geNorm (see Materials and Methods). This analysis indicated that for each experimental condition the inclusion of the best two reference targets is sufficient for adequate normalization as indicated by V_2/3_ values below 0.15 (Figure S4, 0.15 threshold according to Vandesompele *et al.* (2002) [7]). In 5 out of 6 conditions the best two reference targets were EREs.

**Figure 3:**
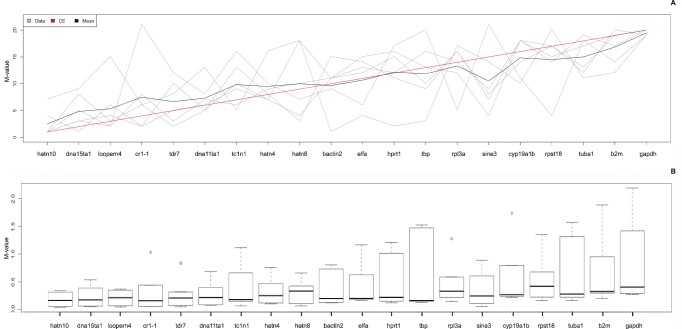
Rank aggregation analysis. **A:** Rank aggregation analysis ordering the reference genes, based on their rank position according to each stability measurement (grey lines), from the most stable (left) to the least stable (right). Mean rank position of each gene is shown in black, as well the model computed by the Monte Carlo algorithm (red line). All EREs, except for *sine3*, are ranked better than the commonly used reference genes. **B:** Box plot representation of dispersion of the M-value. Boxes depict first and third quartile and the median is indicated with a line in the middle of the box, outliers are drawn as circles. Reference targets are ranked according to rank aggregation outcome (most stable reference targets on the left).

Finally, we aimed to identify the most stably expressed reference targets throughout the different experiments performed. A rank aggregation method based on voting theory (Borda count) was used to combine the 6 ranked lists of reference targets, generated for the 6 different experiments. This method tries to find an ordered list of reference assays as close as possible to all individual ordered lists by calculating the weighted Spearman’s footrule distance, and using a cross-entropy Monte Carlo algorithm or genetic algorithm. The analysis of the 6 ordered reference target lists, clearly demonstrated that most of the EREs showed a higher overall expression stability compared to most of the commonly used reference genes, as evidenced by lower ranks and by the lower median M-value (Student’s t-test; p<0.001) and smaller spread of the M-value (Student’s t-test; p<0.001) (Figure 3A,B), with the highest stability for ERE *hatn10*. In each of the 6 experiments, *hatn10* had an M-value below 0.5 and this ERE was found to be the most stably expressed reference target in 4 out of 6 experiments, indicating that *hatn10* is an interesting candidate for inclusion as a reference target in a broad range of experiments.

### Assessment of the validity of ERE reference targets versus common reference genes to normalize genes of interest

To test the accuracy of qPCR results after normalization with either frequently used reference genes (*gapdh*, *bactin2* and *elfa*) or ERE reference targets (*hatn10*, *dna15ta1* and *loopern4*), the expression of known differentially expressed genes was measured in a diverse set of experimental conditions (developmental time series, different organs, morpholino and compound treatments).

**Figure 4:**
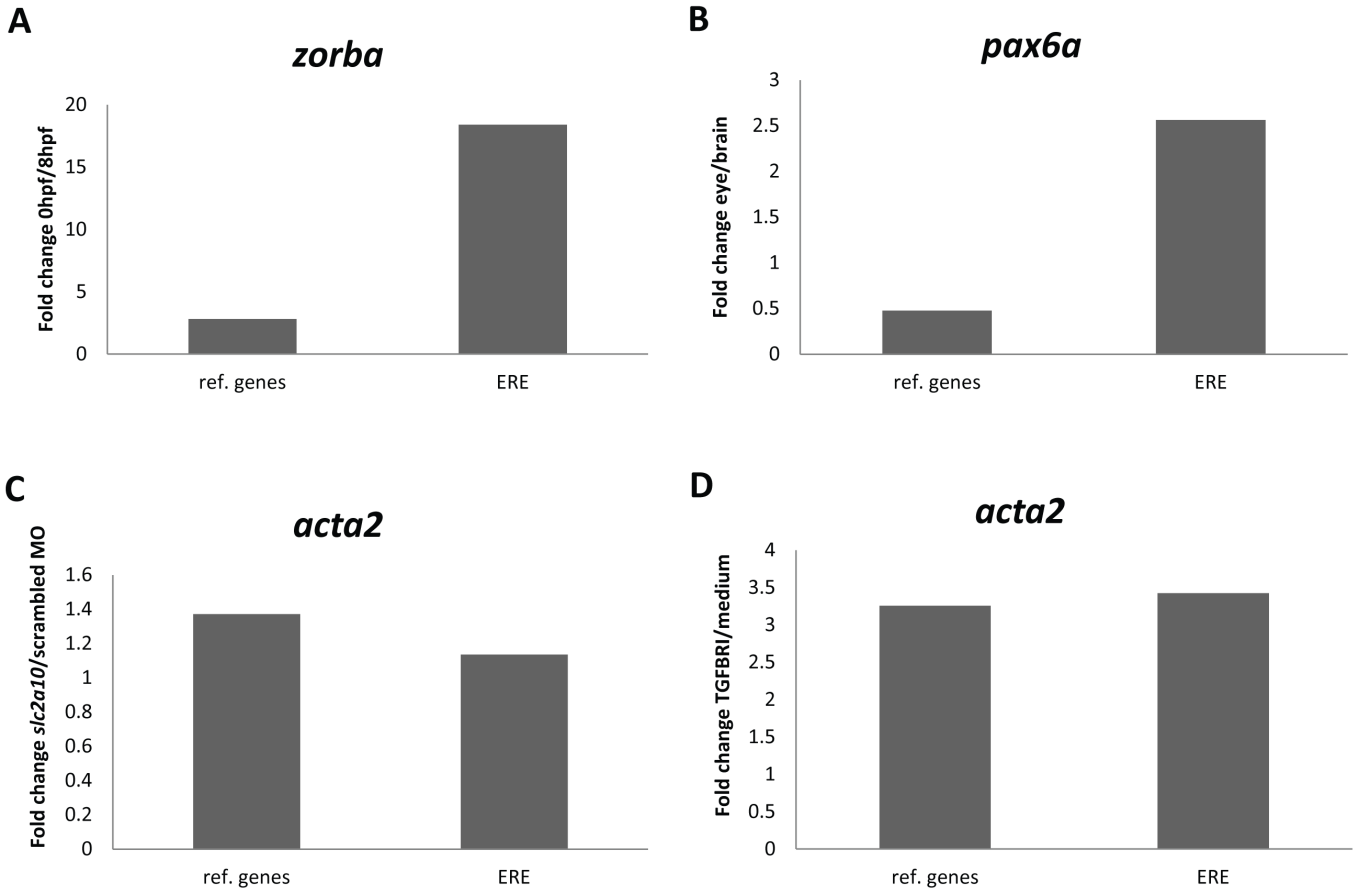
Fold change expression of selected genes of interest after normalization with common reference genes (ref. genes) and with ERE reference targets (ERE). **A:** Fold change expression of *zorba* between 0 hpf and 8 hpf. **B:** Fold change expression of *pax6a* between adult zebrafish eye and brain tissues. **C:** Fold change expression of *acta2* between *slc2a10* MO and scrambled MO injections. **D:** Fold change expression of *acta2* between TGFBRI compound and screening medium treatment.

According to earlier reports, *zorba* transcripts are only present in zebrafish embryos until the mid-blastula transition (MBT) at about 3.5 hpf, after which zygotic transcription is initiated [28], [29]. This means that *zorba* transcripts are strictly maternally derived with almost no zygotic transcription. This was validated by microarray data reported by Yang *et al.* (2013) [30], where transcriptomes were compared between different developmental stages in zebrafish embryos. We looked at *zorba* expression in a developmental time series using RT-qPCR and normalized the data either with frequently used reference genes or with ERE reference targets. When using the ERE’s as reference targets, a more than 20 fold expression difference was noted between the 0 hpf (maternal) and 8 hpf (zygotic) time points, confirming that *zorba* transcripts are almost exclusively maternally derived (Figure 4A). When applying the classic reference genes for normalization, only a threefold expression difference was observed, falsely indicating a relatively small expression difference for *zorba* between maternal and zygotic transcription stages.

During early embryogenesis, the *pax6a* gene is expressed in specific parts of the developing brain, although from larval stages on, expression gets more restricted to the eye [31]. Predominant eye expression of *pax6a* is further evidenced by microarray expression analysis (own data, not shown) revealing a 25% higher *pax6a* expression in the adult zebrafish eye compared to the brain. We looked at *pax6a* RT-qPCR expression levels in different organs from adult zebrafish. When expression levels were normalized to the ERE reference targets, the higher expression of *pax6a* in the eye versus the brain could be confirmed (Figure 4B). In contrast, normalization to the common reference genes resulted in an unexpectedly higher expression of *pax6a* in the brain compared to the eye.

In zebrafish embryos, knockdown of *slc2a10* using MO injection affects the expression of a number of genes involved in cardiovascular development, as evidenced by microarray expression analysis [18]. One of these prototypical affected genes is *acta2*, showing a small upregulation upon *slc2a10* knockdown. We conducted RT-qPCR expression analysis for *acta2* and revealed that both common reference gene and ERE normalization resulted in a similar slight upregulation of the acta2 gene after *slc2a10* MO injection (Figure 4C). The *acta2* gene is also known to be upregulated upon treatment with TGFBRI compound to a greater extent than after *slc2a10* MO injections [18]. We confirmed a threefold overexpression of *acta2* upon administration of TGFBRI compound, both after common reference gene and ERE normalization (Figure 4D).

## Discussion

Several reports indicate that, even within a species, no single gene can be regarded as an ideal reference gene for the normalization of qPCR data across diverse sample types and experimental situations [8], [10], [32]. This is due to variations in expression levels of these genes across different experimental conditions, developmental stages or across different tissues or cells. In this study, we specifically aimed to identify a set of reference targets that are stably expressed over a diverse set of samples obtained from the zebrafish, a model organism which is becoming increasingly popular in disease modeling, developmental studies and toxicology. Our strategy was based on the identification of specific types of repetitive elements that have spread throughout the zebrafish genome during evolution and that are also present in genomic sequences that are transcribed to RNA. With a single pair of RT-qPCR primers, one specific expressed repetitive element (ERE) can be amplified, thereby simultaneously detecting numerous different transcripts in which the specific ERE is present. The underlying assumption is that by measuring many transcripts at the same time, differential expression of a few of them will not drastically alter the total level of ERE expression. Therefore, expression of this set of repeats is expected to be highly stable throughout different experimental situations, as it serves as an estimation of the general mRNA fraction abundance. The use of expressed repeat elements was first presented by Vandesompele *et al.* (2nd International qPCR Symposium, Freising-Weihenstephan, Germany, September 6, 2005) and subsequently confirmed by Marullo *et al.* (2010) [13] where primate specific Alu repeats were used for normalization of biomarkers in human blood. Recently, it has been reported that expressed Alu repeats can be successfully used as a normalization factor in RT-qPCR experiments where human cancer cells were subjected to various perturbations [14] or in human embryonic stem cell differentiation experiments [15].

In this study, 10 different zebrafish EREs were selected as candidate normalization targets based on a minimal number of expressed copies and conservation score. Subsequently, expression stability of these EREs and 10 commonly used reference mRNAs for zebrafish studies were compared. The standard reference genes are involved in different cellular processes and structures such as metabolism (*hprt1, gapdh*), transcription (*tbp*), translation (*elfa*), cytoskeletal structure (*bactin2, tuba1*), major histocompatibility complex (*b2m*) and steroid biosynthesis (*cyp19a1b*), thus avoiding co-regulation upon different treatments [11], [32], [33]. We did not include the frequently used rRNA transcripts (e.g. 18S and 28S rRNA) into this study. Indeed, while rRNA represents more than 90% of total RNA, it has been shown that the rRNA to mRNA ratio can vary depending on the experimental condition [34]–[36]. Moreover, the high abundance of rRNA compared to mRNA may hamper the correction of the baseline fluorescence in qPCR data analysis [7], [37]. Finally, rRNA is transcribed by a different endogenous RNA polymerase, is not polyadenylated, and has a different function compared to mRNA, making ribosomal RNA a non-representative form of RNA for normalization of mRNA. Therefore, the use of rRNA as a normalization factor in qPCR experiments is not recommended and could lead to false interpretation of the data.

Expression stabilities were tested in a diverse sample set, covering different experimental setups in zebrafish research, including morpholino and compound treated samples and samples from different developmental stages and from different adult tissues. Especially for the latter two sample types, good quality normalization factors are difficult to find [11], most likely because of dramatic changes in expression profiles during zebrafish development and major differences in expression between different matured organs [30], [38]. Indeed, expression analysis in different developmental stages and tissues from zebrafish, revealed a poor expression stability of all commonly used reference mRNAs with M-values higher than 0.5, implying that these genes are not suitable for reliable normalization of expression data in these experimental conditions. Strikingly, the expression of one of the most frequently used reference genes, *gapdh*, is the least stable of all reference targets tested in this study. In keeping with this observation, previous studies in vertebrate tissues and cell lines have already reported on the poor performance of *gapdh* as an internal reference gene and on its expression variability [39]–[43]. Consequently, we would strongly discourage further use of *gapdh* as reference gene for normalization in zebrafish experiments. Remarkably, most of the zebrafish EREs performed very well, with in many cases M-values below 0.5, signifying a high expression stability, thus clearly marking EREs as the reference target of choice in these experimental conditions. The robustness of ERE normalization for expression analysis in different developmental stages and tissues from zebrafish was further evidenced by the validation of known differential expression levels for respectively the *zorba* and *pax6a* genes. Normalization with common reference genes resulted in completely different expression patterns, leading to false interpretation of the data. The performance of EREs in terms of stability is less pronounced in perturbation experiments such as compound treatments or morpholino injections. While almost all reference targets scored relatively well, again expression stability of the EREs was generally better than for the common reference genes. The relatively good performance of all reference targets, regardless of their nature, in compound and morpholino experiments reflects the more subtle impact of these treatments on the general expression profile in zebrafish embryos. Indeed, validation of known differential expression levels for the *acta2* gene in these conditions revealed no major difference between both normalization strategies.

To identify the most stably expressed reference targets throughout all different experiments performed, we conducted a rank aggregation analysis. This analysis indicates that the expression stability of the EREs was better than for the common reference genes. ERE *hatn10*, *dna15ta1* and *loopern4* represent the most stable reference targets with M-values ≤0.5 in all 6 experiments. We recommend including at least these 3 genes in zebrafish gene expression studies for evaluation of their suitability as normalization targets.

The MIQE guidelines from 2009 emphasize the need for accurate normalization of RT-qPCR data in order to obtain reliable expression data. However, a recent paper in Nature Methods that surveyed 1700 publications with qPCR-based data from 2009 to 2013 reported the poor application of these guidelines including inadequate normalization procedures with widespread use of single, unvalidated reference genes [44]. It has long been recognized that this can lead to unreliable results, in particular for measuring subtle differences in expression levels. Our study fully complies with the MIQE guidelines and tackles the issue of proper normalization in zebrafish expression studies, by providing for the first time a set of robust candidate reference targets to normalize RT-qPCR data in a wide range of zebrafish experiments. EREs have the potential to dramatically facilitate and improve gene expression studies in zebrafish. In addition, the bio-informatics strategy outlined for identification and validation of such EREs in this study can be applied to other organisms. As such, we expect similar ERE qPCR assays to be developed and used in other model organisms for normalization purposes.

## Supporting Information

Figure S1 **Representative example of an ERE standard dilution and melting curve.** **A:** Standard dilution curve, used to determine the primer amplification efficiency of the *dna15ta1* primer set. In this example Cq values obtained for the *dna15ta1* primer set are plotted against the cDNA quantity (ng) (exported from qbase+ software). For each quantity two technical replicates are included. **B:** Melting curve analysis for the *dna15ta1* primer set (exported from LightCycler 480 software). On top, the sample fluorescence is plotted against temperature. Below, the first negative derivative of the sample fluorescence is plotted against temperature, displaying the melting temperature as a peak. In this example, there is a single sharp peak from an amplicon having a Tm of 76°C, indicating the specificity of the *dna15ta1* primer set.
(DOCX)

Figure S2 **Schematic representation of ERE primer design (hypothetical example).** The full-length repeat element (dark grey line, top) and a number of aligned repeat element containing fragments obtained from a combined RefSeq/non-RefSeq blastn search are depicted. In a first step we determine the part of the ERE sequence that is most frequently expressed. To delineate this area, all RefSeq and non-RefSeq blast results are aligned with the consensus repeat sequence and sequences that are commonly present in most of the fragments are used as a template for primer design using primer 3 with default settings.
(TIF)

Figure S3 **Average expression stability of common reference genes and expressed repeat elements (based on LinRegPCR corrected Cq values).**
(TIF)

Figure S4 **GeNorm calculated pairwise variation Vn/n+1 values for the different experimental conditions.** The optimal number of reference targets (n) is reached, when the inclusion of the next reference target (n+1) reduces the Vn/n+1 value below 0.15. For every experiment the V2/3 value is lower than 0.15, indicating that the inclusion of only two reference targets, the ones with the lowest M-value, is sufficient for adequate normalization.
(TIF)

Table S1 **Target specific amplification efficiency parameters.**
(DOCX)

## References

[1] Bustin SA, Benes V, Garson JA, Hellemans J, Huggett J, et al (2009) The MIQE guidelines: minimum information for publication of quantitative real-time PCR experiments. Clin Chem 55: 611–622. 19246619 10.1373/clinchem.2008.112797

[2] Derveaux S, Vandesompele J, Hellemans J (2010) How to do successful gene expression analysis using real-time PCR. Methods 50: 227–230. 19969088 10.1016/j.ymeth.2009.11.001

[3] Dheda K, Huggett JF, Chang JS, Kim LU, Bustin SA, et al (2005) The implications of using an inappropriate reference gene for real-time reverse transcription PCR data normalization. Anal Biochem 344: 141–143. 16054107 10.1016/j.ab.2005.05.022

[4] Goossens K, Van Poucke M, Van Soom A, Vandesompele J, Van Zeveren A, et al (2005) Selection of reference genes for quantitative real-time PCR in bovine preimplantation embryos. BMC Dev Biol 5: 27. 16324220 10.1186/1471-213X-5-27 PMC1315359

[5] Kim BS, Rha SY, Cho GB, Chung HC (2004) Spearman's footrule as a measure of cDNA microarray reproducibility. Genomics 84: 441–448. 15234007 10.1016/j.ygeno.2004.02.015

[6] Tricarico C, Pinzani P, Bianchi S, Paglierani M, Distante V, et al (2002) Quantitative real-time reverse transcription polymerase chain reaction: normalization to rRNA or single housekeeping genes is inappropriate for human tissue biopsies. Anal Biochem 309: 293–300. 12413463 10.1016/s0003-2697(02)00311-1

[7] Vandesompele J, De Preter K, Pattyn F, Poppe B, Van Roy N, et al (2002) Accurate normalization of real-time quantitative RT-PCR data by geometric averaging of multiple internal control genes. Genome Biol. 3: RESEARCH0034. 10.1186/gb-2002-3-7-research0034 PMC126239 12184808

[8] Dhorne-Pollet S, Thelie A, Pollet N (2013) Validation of novel reference genes for RT-qPCR studies of gene expression in Xenopus tropicalis during embryonic and post-embryonic development. Dev Dyn 242: 709–717. 23559567 10.1002/dvdy.23972

[9] Jacob F, Guertler R, Naim S, Nixdorf S, Fedier A, et al (2013) Careful selection of reference genes is required for reliable performance of RT-qPCR in human normal and cancer cell lines. PLoS One 8: e59180. 23554992 10.1371/journal.pone.0059180 PMC3598660

[10] Ledderose C, Heyn J, Limbeck E, Kreth S (2011) Selection of reliable reference genes for quantitative real-time PCR in human T cells and neutrophils. BMC Res Notes 4: 427. 22011438 10.1186/1756-0500-4-427 PMC3229292

[11] McCurley AT, Callard GV (2008) Characterization of housekeeping genes in zebrafish: male-female differences and effects of tissue type, developmental stage and chemical treatment. BMC Mol Biol 9: 102. 19014500 10.1186/1471-2199-9-102 PMC2588455

[12] Hellemans J, Mortier G, De Paepe A, Speleman F, Vandesompele J (2007) qBase relative quantification framework and software for management and automated analysis of real-time quantitative PCR data. Genome Biol 8: R19. 17291332 10.1186/gb-2007-8-2-r19 PMC1852402

[13] Marullo M, Zuccato C, Mariotti C, Lahiri N, Tabrizi SJ, et al (2010) Expressed Alu repeats as a novel, reliable tool for normalization of real-time quantitative RT-PCR data. Genome Biol. 11: R9. 10.1186/gb-2010-11-1-r9 PMC2847721 20109193

[14] Rihani A, Van Maerken T, Pattyn F, Van Peer G, Beckers A, et al (2013) Effective Alu repeat based RT-Qpcr normalization in cancer cell perturbation experiments. PLoS One 8: e71776. 23977142 10.1371/journal.pone.0071776 PMC3743747

[15] Vossaert L, O'Leary T, Van Neste C, Heindryckx B, Vandesompele J, et al (2013) Reference loci for RT-qPCR analysis of differentiating human embryonic stem cells. BMC Mol Biol 14: 21. 24028740 10.1186/1471-2199-14-21 PMC3848990

[16] Nasevicius A, Ekker SC (2000) Effective targeted gene knockdown' in zebrafish. Nat Genet 26: 216–220. 11017081 10.1038/79951

[17] Coucke PJ, Willaert A, Wessels MW, Callewaert B, Zoppi N, et al (2006) Mutations in the facilitative glucose transporter GLUT10 alter angiogenesis and cause arterial tortuosity syndrome. Nat Genet 38: 452–457. 16550171 10.1038/ng1764

[18] Willaert A, Khatri S, Callewaert BL, Coucke PJ, Crosby SD, et al (2012) GLUT10 is required for the development of the cardiovascular system and the notochord and connects mitochondrial function to TGFbeta signaling. Hum Mol Genet 21: 1248–1259. 22116938 10.1093/hmg/ddr555 PMC3284116

[19] Rojas JC, Aguilar B, Rodriguez-Maldonado E, Collados MT (2005) Pharmacogenetics of oral anticoagulants. Blood Coagul Fibrinolysis 16: 389–398. 16093729 10.1097/01.mbc.0000174079.47248.0c

[20] Weigt S, Huebler N, Strecker R, Braunbeck T, Broschard TH (2012) Developmental effects of coumarin and the anticoagulant coumarin derivative warfarin on zebrafish (Danio rerio) embryos. Reprod Toxicol 33: 133–141. 21798343 10.1016/j.reprotox.2011.07.001

[21] Murphey RD, Zon LI (2006) Small molecule screening in the zebrafish. Methods 39: 255–261. 16877005 10.1016/j.ymeth.2005.09.019

[22] Gupta T, Mullins MC (2010) Dissection of organs from the adult zebrafish. J Vis Exp. 10.3791/1717 PMC3144575 20203557

[23] Vermeulen J, Derveaux S, Lefever S, De Smet E, De Preter K, et al (2009) RNA pre-amplification enables large-scale RT-qPCR gene-expression studies on limiting sample amounts. BMC Res Notes 2: 235. 19930725 10.1186/1756-0500-2-235 PMC2789097

[24] Untergasser A, Cutcutache I, Koressaar T, Ye J, Faircloth BC, et al (2012) Primer3–new capabilities and interfaces. Nucleic Acids Res 40: e115. 22730293 10.1093/nar/gks596 PMC3424584

[25] Ruijter JM, Ramakers C, Hoogaars WM, Karlen Y, Bakker O, et al (2009) Amplification efficiency: linking baseline and bias in the analysis of quantitative PCR data. Nucleic Acids Res 37: e45. 19237396 10.1093/nar/gkp045 PMC2665230

[26] Pihur V, Datta S, Datta S (2009) RankAggreg, an R package for weighted rank aggregation. BMC Bioinformatics 10: 62. 19228411 10.1186/1471-2105-10-62 PMC2669484

[27] Jurka J, Kapitonov VV, Pavlicek A, Klonowski P, Kohany O, et al (2005) Repbase Update, a database of eukaryotic repetitive elements. Cytogenet Genome Res 110: 462–467. 16093699 10.1159/000084979

[28] Bally-Cuif L, Schatz WJ, Ho RK (1998) Characterization of the zebrafish Orb/CPEB-related RNA binding protein and localization of maternal components in the zebrafish oocyte. Mech Dev 77: 31–47. 9784598 10.1016/s0925-4773(98)00109-9

[29] Kimmel CB, Ballard WW, Kimmel SR, Ullmann B, Schilling TF (1995) Stages of embryonic development of the zebrafish. Dev Dyn 203: 253–310. 8589427 10.1002/aja.1002030302

[30] Yang H, Zhou Y, Gu J, Xie S, Xu Y, et al (2013) Deep mRNA sequencing analysis to capture the transcriptome landscape of zebrafish embryos and larvae. PLoS One 8: e64058. 23700457 10.1371/journal.pone.0064058 PMC3659048

[31] Lakowski J, Majumder A, Lauderdale JD (2007) Mechanisms controlling Pax6 isoform expression in the retina have been conserved between teleosts and mammals. Dev Biol 307: 498–520. 17509554 10.1016/j.ydbio.2007.04.015

[32] Casadei R, Pelleri MC, Vitale L, Facchin F, Lenzi L, et al (2011) Identification of housekeeping genes suitable for gene expression analysis in the zebrafish. Gene Expr Patterns 11: 271–276. 21281742 10.1016/j.gep.2011.01.003

[33] Tang R, Dodd A, Lai D, McNabb WC, Love DR (2007) Validation of zebrafish (Danio rerio) reference genes for quantitative real-time RT-PCR normalization. Acta Biochim Biophys Sin (Shanghai). 39: 384–390. 10.1111/j.1745-7270.2007.00283.x PMC7110012 17492136

[34] Hansen MC, Nielsen AK, Molin S, Hammer K, Kilstrup M (2001) Changes in rRNA levels during stress invalidates results from mRNA blotting: fluorescence in situ rRNA hybridization permits renormalization for estimation of cellular mRNA levels. J Bacteriol 183: 4747–4751. 11466277 10.1128/JB.183.16.4747-4751.2001 PMC99528

[35] Huggett J, Dheda K, Bustin S, Zumla A (2005) Real-time RT-PCR normalisation; strategies and considerations. Genes Immun. 6: 279–284. 10.1038/sj.gene.6364190 15815687

[36] Solanas M, Moral R, Escrich E (2001) Unsuitability of using ribosomal RNA as loading control for Northern blot analyses related to the imbalance between messenger and ribosomal RNA content in rat mammary tumors. Anal Biochem 288: 99–102. 11141312 10.1006/abio.2000.4889

[37] Hendriks-Balk MC, Michel MC, Alewijnse AE (2007) Pitfalls in the normalization of real-time polymerase chain reaction data. Basic Res Cardiol 102: 195–197. 17370033 10.1007/s00395-007-0649-0 PMC2779446

[38] Abramsson A, Westman-Brinkmalm A, Pannee J, Gustavsson M, von Otter M, et al (2010) Proteomics profiling of single organs from individual adult zebrafish. Zebrafish 7: 161–168. 20392139 10.1089/zeb.2009.0644

[39] Barber RD, Harmer DW, Coleman RA, Clark BJ (2005) GAPDH as a housekeeping gene: analysis of GAPDH mRNA expression in a panel of 72 human tissues. Physiol Genomics 21: 389–395. 15769908 10.1152/physiolgenomics.00025.2005

[40] Bustin SA (2000) Absolute quantification of mRNA using real-time reverse transcription polymerase chain reaction assays. J Mol Endocrinol 25: 169–193. 11013345 10.1677/jme.0.0250169

[41] Bustin SA (2002) Quantification of mRNA using real-time reverse transcription PCR (RT-PCR): trends and problems. J Mol Endocrinol. 29: 23–39. 10.1677/jme.0.0290023 12200227

[42] Dheda K, Huggett JF, Bustin SA, Johnson MA, Rook G, et al. (2004) Validation of housekeeping genes for normalizing RNA expression in real-time PCR. Biotechniques 37: 112–114, 116, 118–119. 10.2144/04371RR03 15283208

[43] Lin J, Redies C (2012) Histological evidence: housekeeping genes beta-actin and GAPDH are of limited value for normalization of gene expression. Dev Genes Evol 222: 369–376. 23099774 10.1007/s00427-012-0420-x

[44] Bustin SA, Benes V, Garson J, Hellemans J, Huggett J, et al (2013) The need for transparency and good practices in the qPCR literature. Nat Methods 10: 1063–1067. 24173381 10.1038/nmeth.2697

