# Supplementary figures and images for: Expressed Repeat Elements Improve RT-qPCR Normalization across a Wide Range of Zebrafish Gene Expression Studies
Source: PLoS One. 2014 Oct 13;9(10):e109091. doi: 10.1371/journal.pone.0109091 (PMC4195698; doi:10.1371/journal.pone.0109091)

A


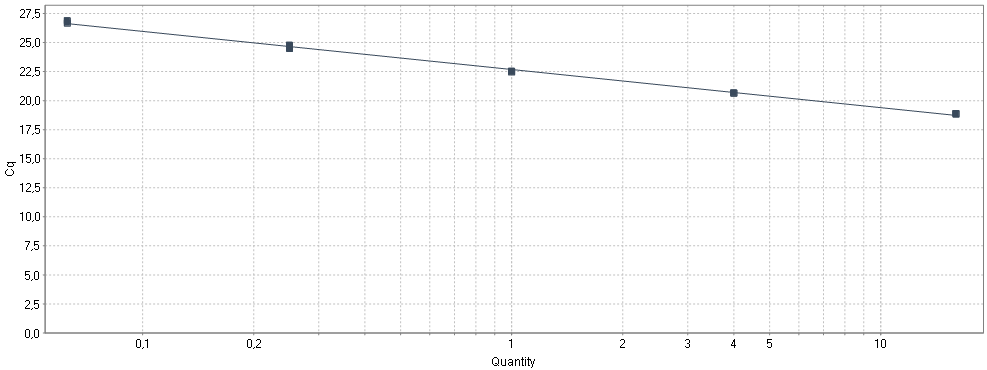


B


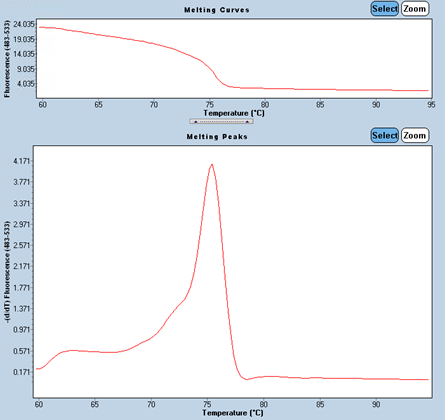

Supplement: Figure S1 — Representative example of an ERE standard dilution and melting curve. A: Standard dilution curve, used to determine the primer amplification efficiency of the dna15ta1 primer set. In this example Cq values obtained for the dna15ta1 primer set are plotted against the cDNA quantity (ng) (exported from qbase+ software). For each quantity two technical replicates are included. B: Melting curve analysis for the dna15ta1 primer set (exported from LightCycler 480 software). On top, the sample fluorescence is plotted against temperature. Below, the first negative derivative of the sample fluorescence is plotted against temperature, displaying the melting temperature as a peak. In this example, there is a single sharp peak from an amplicon having a Tm of 76°C, indicating the specificity of the dna15ta1 primer set. (DOCX) [file pone.0109091.s001.docx]

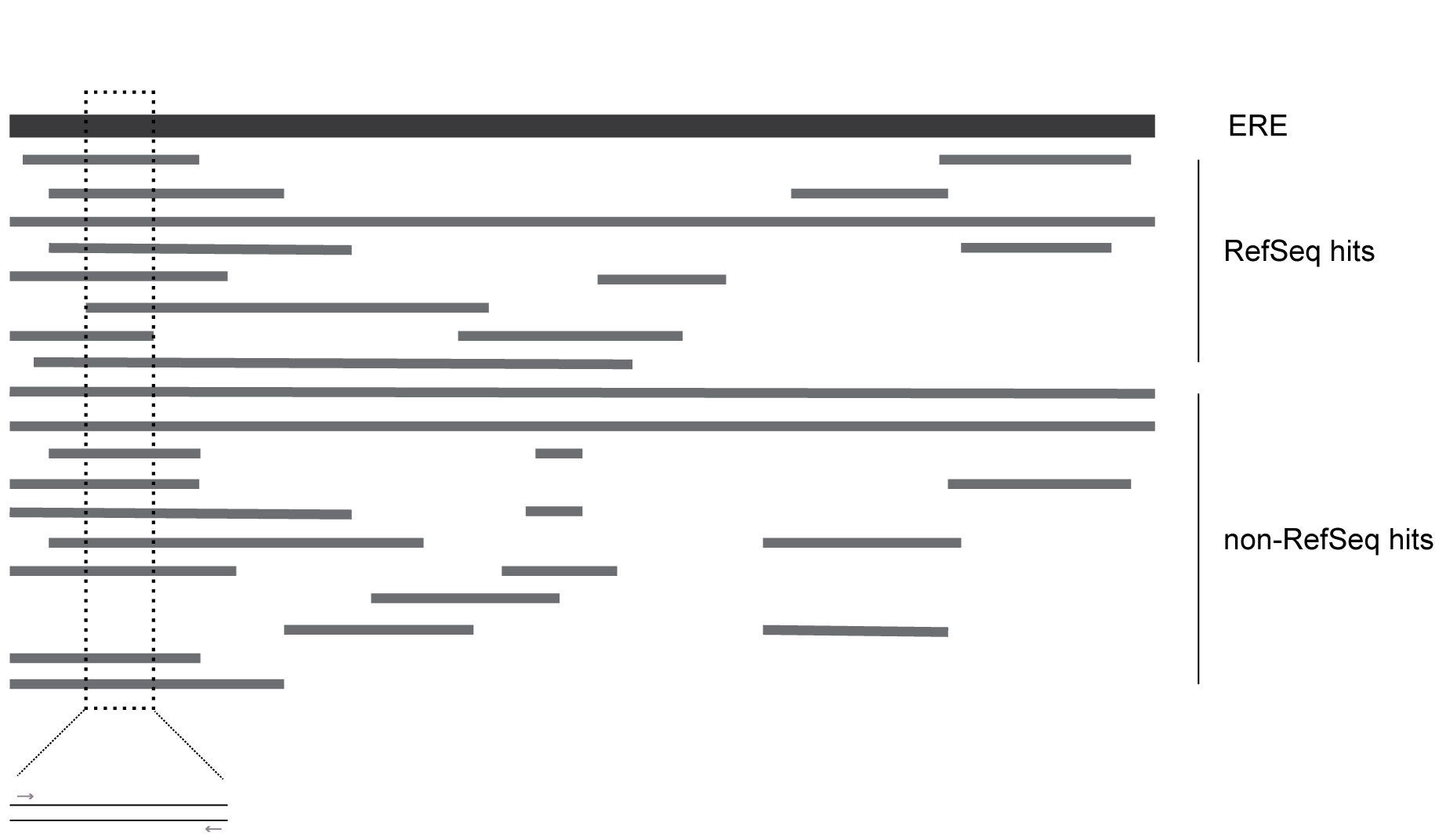

Supplement: Figure S2 — Schematic representation of ERE primer design (hypothetical example). The full-length repeat element (dark grey line, top) and a number of aligned repeat element containing fragments obtained from a combined RefSeq/non-RefSeq blastn search are depicted. In a first step we determine the part of the ERE sequence that is most frequently expressed. To delineate this area, all RefSeq and non-RefSeq blast results are aligned with the consensus repeat sequence and sequences that are commonly present in most of the fragments are used as a template for primer design using primer 3 with default settings. (TIF) [file pone.0109091.s002.tif]

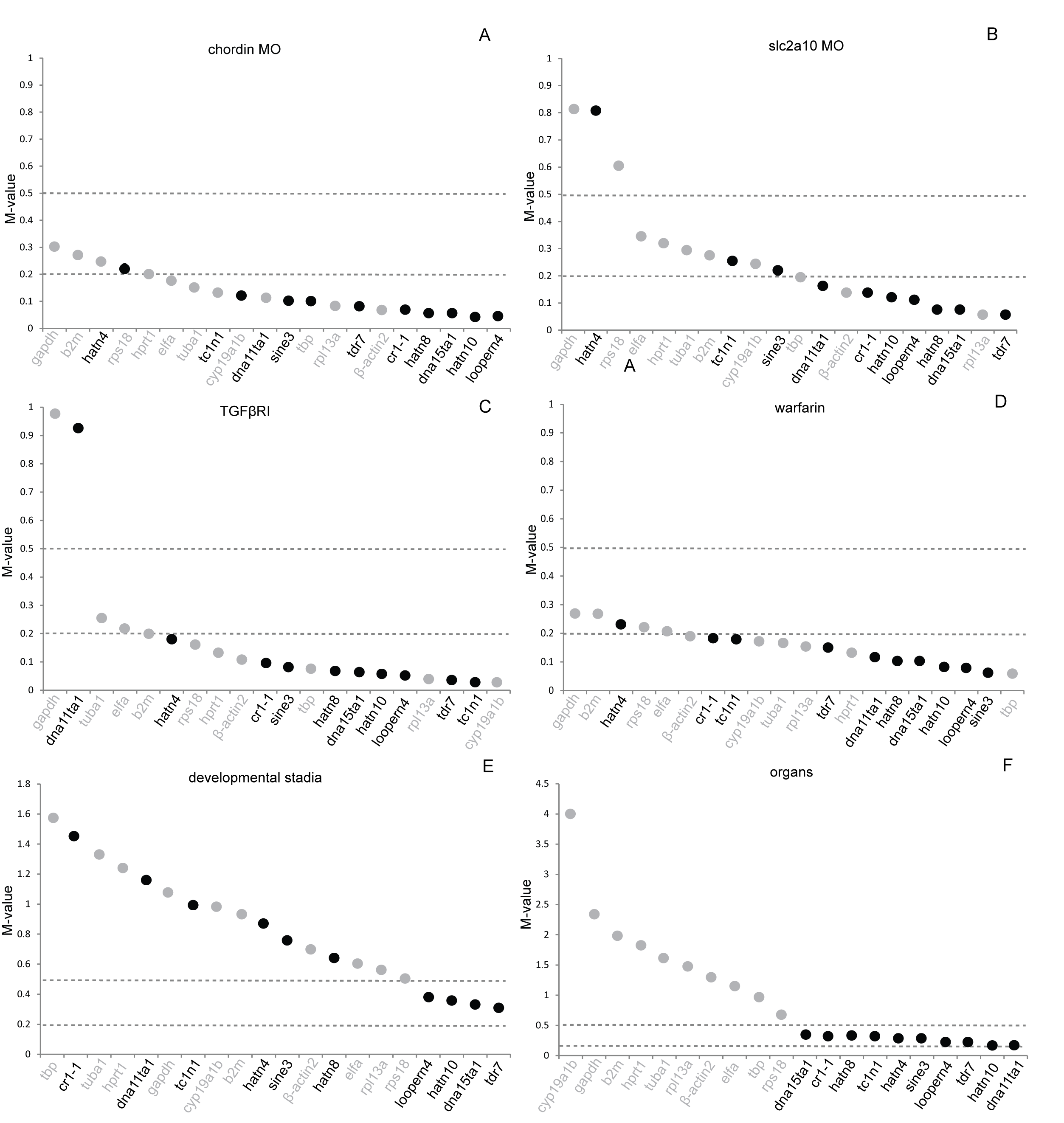

Supplement: Figure S3 — Average expression stability of common reference genes and expressed repeat elements (based on LinRegPCR corrected Cq values). (TIF) [file pone.0109091.s003.tif]

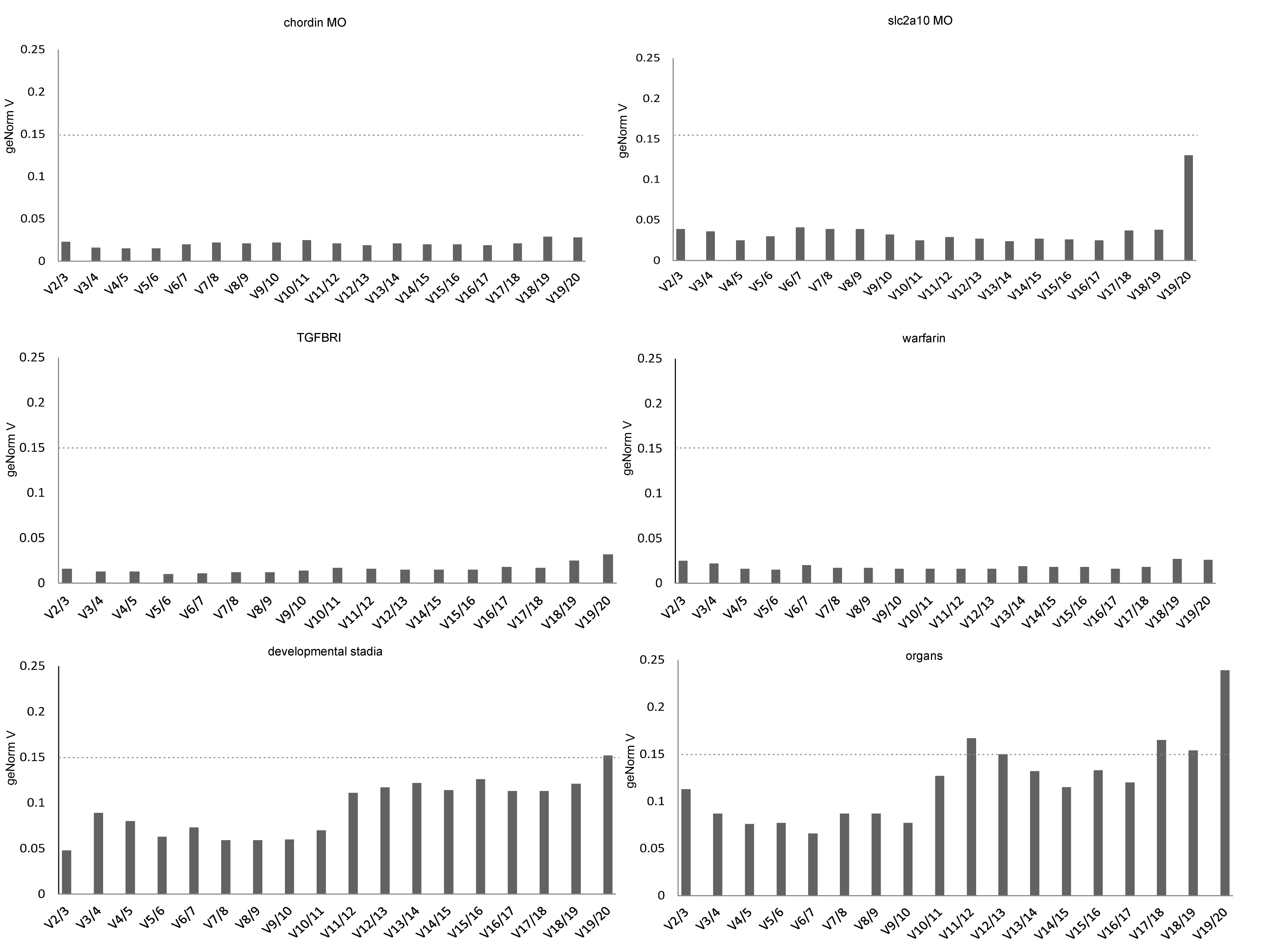

Supplement: Figure S4 — GeNorm calculated pairwise variation Vn/n+1 values for the different experimental conditions. The optimal number of reference targets (n) is reached, when the inclusion of the next reference target (n+1) reduces the Vn/n+1 value below 0.15. For every experiment the V2/3 value is lower than 0.15, indicating that the inclusion of only two reference targets, the ones with the lowest M-value, is sufficient for adequate normalization. (TIF) [file pone.0109091.s004.tif]
