# Supplementary material for: Expressed Repeat Elements Improve RT-qPCR Normalization across a Wide Range of Zebrafish Gene Expression Studies
Source: PLoS One. 2014 Oct 13;9(10):e109091. doi: 10.1371/journal.pone.0109091 (PMC4195698; doi:10.1371/journal.pone.0109091)
Supplement: Table S1 — Target specific amplification efficiency parameters. (DOCX) [file pone.0109091.s005.docx]

| **Reference target** | **E computed** | **E (SE) computed** | **R^2^ computed** | **Slope computed** | **Efficiency (%)** | **Efficiency LinRegPCR** |
| --- | --- | --- | --- | --- | --- | --- |
| *cr1-1* | 2.012 | 0.018 | 0.999 | -3.293 | 101.22 | 1.85 |
| *dna11ta1* | 2.076 | 0.04 | 0.994 | -3.153 | 107.57 | 1.89 |
| *dna15ta1* | 2.012 | 0.027 | 0.997 | -3.295 | 101.14 | 1.94 |
| *hatn10* | 2.043 | 0.013 | 1 | -3.224 | 104.25 | 1.88 |
| *hatn4* | 2.05 | 0.02 | 0.999 | -3.208 | 104.98 | 1.87 |
| *hatn8* | 2.025 | 0.027 | 0.997 | -3.265 | 102.43 | 1.84 |
| *loopern4* | 2.095 | 0.035 | 0.996 | -3.114 | 109.47 | 1.87 |
| *sine3* | 2.014 | 0.036 | 0.996 | -3.288 | 101.44 | 1.86 |
| *tc1n1* | 2.034 | 0.049 | 0.993 | -3.242 | 103.45 | 1.88 |
| *tdr7* | 2.022 | 0.036 | 0.996 | -3.271 | 102.17 | 1.87 |
| *b2m* | 1.946 | 0.057 | 0.992 | -3.459 | 94.6 | 1.86 |
| *bactin2* | 1.993 | 0.054 | 0.991 | -3.340 | 99.3 | 1.86 |
| *cyp19a1b* | 2.015 | 0.045 | 0.994 | -3.288 | 101.5 | 1.84 |
| *elfa* | 2.060 | 0.049 | 0.993 | -3.187 | 106.0 | 1.88 |
| *hprt1* | 1.957 | 0.042 | 0.994 | -3.430 | 95.7 | 1.86 |
| *rpl13a* | 1.912 | 0.040 | 0.994 | -3.553 | 91.2 | 1.87 |
| *rps18* | 1.987 | 0.042 | 0.994 | -3.352 | 98.7 | 1.86 |
| *tbp* | 1.954 | 0.055 | 0.990 | -3.438 | 95.4 | 1.85 |
| *tuba1* | 2.077 | 0.024 | 0.998 | -3.150 | 107.7 | 1.89 |
| *gapdh* | 2.025 | 0.07 | 0.976 | -3.263 | 102.5 | 1.89 |
